# Reverse and antegrade vector thread lifting techniques: Correcting nasolabial and marionette lines

**DOI:** 10.1111/jocd.16520

**Published:** 2024-08-06

**Authors:** Soo Yeon Park, Soo‐Bin Kim, Jovian Wan, Kashif Ali Samin, Kar Wai Alvin Lee, Lisa Kwin Wah Chan, Atchima Suwanchinda, Kyu‐Ho Yi

**Affiliations:** ^1^ Made‐Young Plastic Surgery Clinic Seoul Korea; ^2^ Division in Anatomy and Developmental Biology, Department of Oral Biology Human Identification Research Institute, BK21 FOUR Project, Yonsei University College of Dentistry Seoul Korea; ^3^ Asia Pacific Aesthetic Academy Hong Kong Hong Kong; ^4^ Department of Aesthetic Medicine Khyber Medical University Peshawar Pakistan; ^5^ EverKeen Medical Centre Hong Kong Hong Kong; ^6^ Department of Dermatology, Chulabhorn hospital Chulabhorn Royal Academy Bangkok Thailand; ^7^ Department of Dermatology Chulabhorn International Collage of Medicine, Thammasat University Bangkok Thailand; ^8^ Division of Dermatology, Department of Medicine, Faculty of Medicine, Ramathibodi Hospital Mahidol University Bangkok Thailand; ^9^ Maylin Clinic (Apgujeong) Seoul Korea

**Keywords:** marionette line, nasolabial fold, polydioxanone threads, reversal technique, thread lifting

## Abstract

**Background:**

This study evaluates the efficacy of reverse and antegrade thread lifting vectors in conjunction with thread selection for correcting nasolabial folds and marionette lines, aiming to enhance treatment precision and effectiveness.

**Methods:**

Three female patients aged 43, 48, and 53, presenting with primary concerns regarding nasolabial folds, underwent distinct treatment regimens utilizing various types of threads and vectors. Additionally, video demonstrations were recorded to showcase the procedural techniques employed in each case.

**Results:**

The outcomes of the thread lifting procedures demonstrated significant improvements in the correction of the nasolabial fold and marionette line.

**Conclusion:**

Innovative thread insertion techniques involve entry points above the zygomatic arch and threading toward the temple hairline for lateral face lifting. These utilize barbs to pull skin and underlying tissues. The reversal technique involves inserting threads in a criss‐cross pattern, forming a fibrous structure that may prolong the duration of results. Various thread combinations, considering factors such as composition, thickness, and barb size, provide customized procedures. This research introduces clinical applications, including temple area hairline lifting for nasolabial fold and marionette line correction, reverse vectors targeting nasolabial folds and marionette lines, and antegrade approaches from the lateral side.

## 1 INTRODUCTION

The selection of threads for lifting procedures is a critical aspect that significantly influences the efficacy and longevity of outcomes. The composition and thickness of these threads play pivotal roles in determining their lifting effects and the duration of maintenance. Historically, non‐absorbable threads containing polypropylene (PP) were widely utilized in such procedures. However, contemporary practices have witnessed a shift toward the predominant use of polydioxanone (PDO) threads due to their recognized effectiveness. Additionally, threads fabricated from materials such as poly‐L‐lactic acid (PLLA) and polycaprolactone (PCL) are also incorporated in certain cases, reflecting the diversification of available options.^1^, ^2^

PDO threads exhibit considerable tensile strength among absorbable threads and are preferred due to their relatively longer duration of degradation, lasting for over 6 months, making them widely utilized. Additionally, the composition of PDO threads influences their elasticity, while their thickness plays a pivotal role in maintaining the tensile strength of lifting threads.^1^, ^3^ Furthermore, in cog threads, the number and size of barbs (cogs) within the thread significantly impact the lifting effects, offering versatility in thread combinations based on the composition, thickness, and the shape and size of the barbs. Optimal thread selection according to the intended procedural goals enables tailored and customized procedures. In the manufacturing process of cog thread products using PDO thread filaments, the initial steps involve creating the cogs, followed by subsequent processes such as attaching them to needles or modifying their shape. These preparations come before the final sterilization and packaging of the product. Upon unsealing the packaged product, exposure to ambient moisture triggers the degradation process. Though not immediately visible to the naked eye, threads gradually degrade upon contact with moisture. In clinical scenarios, the use of older, previously unsealed products may result in thread breakage due to degradation, which starts shortly after opening. For these reasons, notwithstanding the similarity in the quality of the thread filament, products subjected to less stringent processing may have encountered exposure to ambient moisture during the final stages of manufacturing. Consequently, both the immediate efficacy and the longevity of the thread may unavoidably diminish.

**FIGURE 1:**
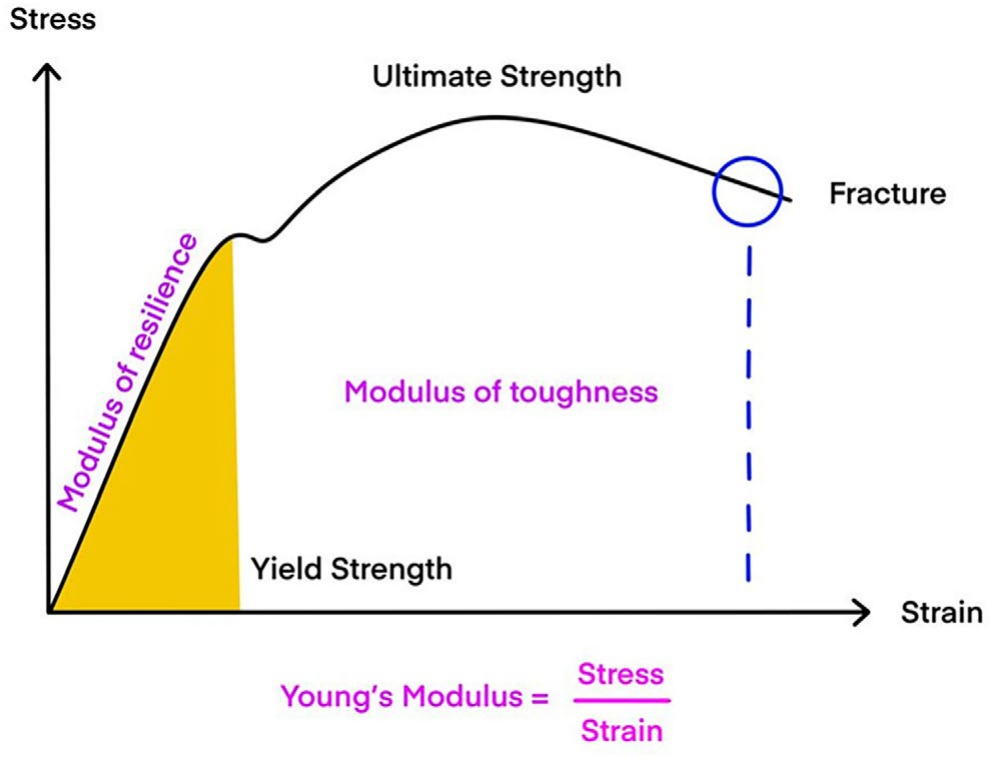
Young's modulus, also called the elastic modulus, is a measure that signifies how much an elastic object's length changes in response to applied stress.

Young's modulus, also known as the elastic modulus, represents the coefficient indicating how the relative length of an elastic object changes concerning applied stress (Figure 1).^4^ High modulus indicates a material that is rigid and less compressible, possessing significant restorative capabilities. Understanding the elasticity inherent in each thread enables the creation of harmonious results by skillfully mixing threads tailored to individual patients. The modulus is not solely determined by the composition of threads such as PDO, PLLA, or PCL; rather, it can also vary within PDO threads themselves depending on the manufacturing process.^5^, ^6^, ^7^, ^8^, ^9^, ^10^ Employing threads with a high modulus may initially produce excellent lifting effects but could trigger a foreign body sensation when muscles or tissues move. Conversely, using threads with a low modulus minimizes discomfort during facial movements or expressions but may result in a weaker lifting effect. The longevity of the thread lifting procedure relies on the anchoring and fixation sites, as well as the material composition of the thread utilized.

The nasolabial folds, known as the skin creases extending bilaterally from the nasal wings to the corners of the mouth, are defined by facial structures supporting the buccal fat pad, creating a distinct boundary between the cheeks and the upper lip. The term originates from the Latin “nasus,” referring to the nose, and “labium,” meaning the lip. It is important to note that the accurate anatomical term for this fold is the melolabial fold, representing the crease between the cheek and the lip. As individuals age naturally, these folds may undergo an increase in both length and depth.^11^, ^12^

Marionette lines, also known as melomental folds, are facial wrinkles that develop as a result of aging, appearing as downward‐curving lines starting from the corners of the mouth. These lines were conventionally linked to repetitive facial expressions and the effects of gravity on less elastic skin tissue. Historically, treating well‐defined marionette lines has been challenging in cosmetic procedures.

Expanding on the foundational principles outlined earlier, this study aims to introduce how thread lifting, using commonly used thread types, can be applied to correct nasolabial folds and marionette lines. The objective is to integrate theoretical frameworks and designs into practical use, thereby enhancing the effectiveness and precision of aesthetic procedures in these facial regions.

## 2 REVERSE TECHNIQUE

In traditional lateral face lifting procedures, the standard practice involved inserting barbed threads near the hairline around the zygomatic area to achieve thread lifting. However, there has been a shift toward the use of barbed threads with a reverse vector in recent times (Videos S1 and S2). This method entails creating an entry point above the zygomatic arch and threading the material inwards toward the temple hairline in the opposite direction. The aim of this technique is to elevate the skin and tissues near the zygomatic arch toward the temple hairline.^13^ This effect is facilitated by the attachment of the barbs to the tissues; when the threads are pulled, lax tissues are drawn towards firmer tissues. Therefore, even when the barbed threads are inserted from below and pulled upwards, it is not the upper tissues descending toward the entry point, but rather the tissues around the entry point being pulled upwards toward the firmer tissues located higher near the zygomatic region.

**FIGURE 2:**
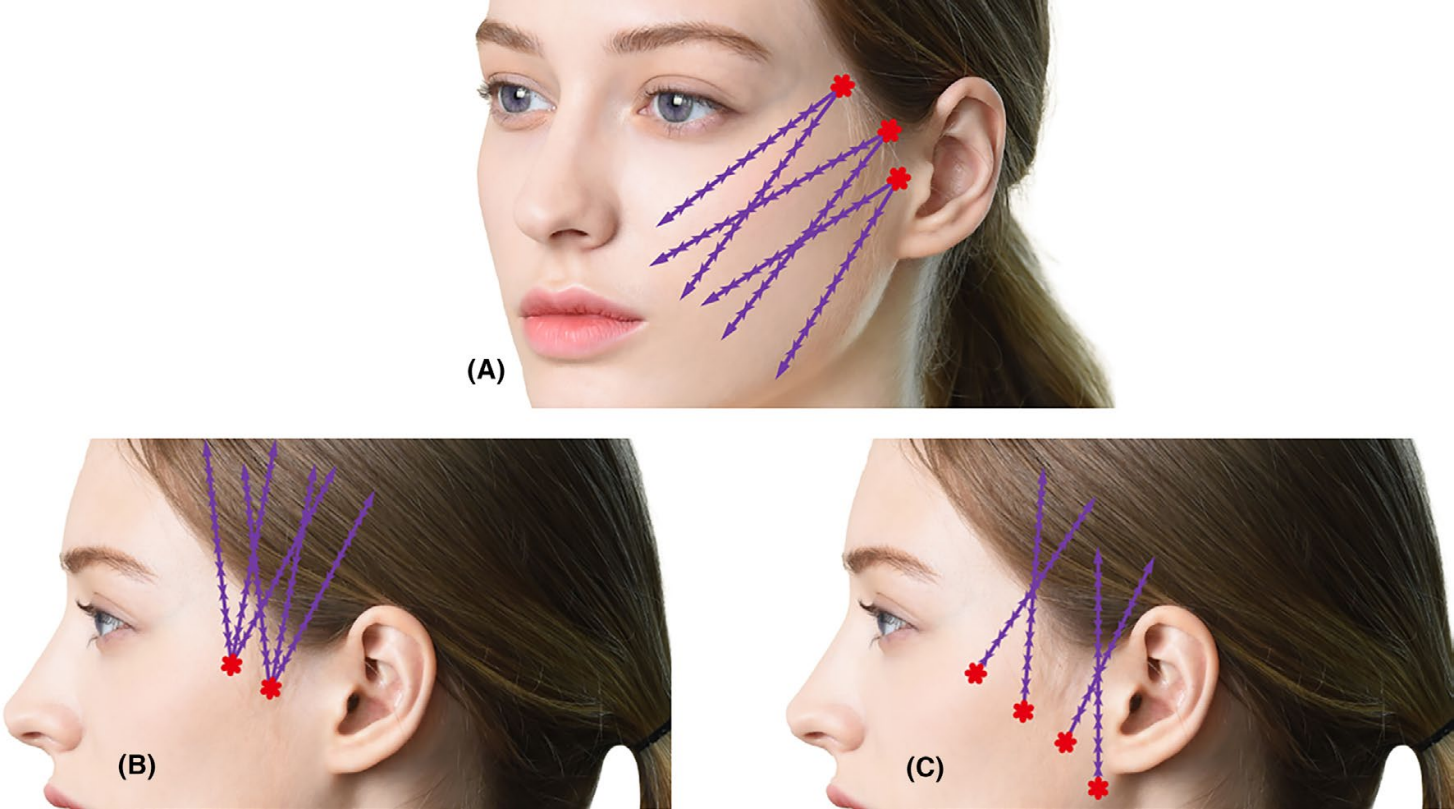
Creating a crisscross pattern during implantation involves utilizing common target points but with different entry points and diverse directions to build an internal grid resembling a fibrous structure (A). This technique aims to ensure that during thread insertion, they intertwine in a crisscross manner, potentially enhancing the lasting impact of the procedure. (B) Multiple floating‐type threads are inserted from a single entry point marked on the zygomatic arch, typically between the sideburn and lateral orbital rim. Avoiding positioning too close to the eye's outer corner is crucial to prevent eyelid skin elevation. (C) Demonstrates an alternative method involving a single floating‐type thread entry, marked along the line connecting the eye's outer corner to the ear. This method involves pulling tissues toward the temple hairline, allowing for upward tissue movement without causing downward pulling, utilizing the superficial temporal fascia to anchor the thread without damaging vascular or neural structures during cannula insertion beneath this space. These techniques are done with Bidirectional thread (Epiticon BI, Jetema Inc., Korea).

When inserting the threads, ensuring that each thread forms a criss‐crossing pattern can create a fibrous architecture, potentially prolonging the longevity of the results significantly (Figure 2A).

## 3 TEMPLE AREA HAIRLINE LIFTING (CORE LIFTING)

Before the procedure, while the patient is seated, mark the entry point for cannula insertion on the zygomatic arch. Typically, this point is located at the midpoint of the zygomatic arch between the anterior margin of the sideburn and the lateral margin of the orbital rim. Avoid marking too close to the outer eye corner to prevent elevating the thin eyelid skin, affecting facial expressions; instead, manually manipulate the skin upward by pressing with fingers to determine the cannulas insertion position and direction.

When marking the entry point, avoid positioning it too close to the inferior aspect of the zygomatic arch, where the fibers of the zygomatico‐cutaneous ligament attach to the arch's inferior margin. Inserting threads into this area, where the skin is strongly tethered by robust fibrous tissues, might not follow the hairline well. Therefore, it is advisable not to place the insertion point too far below the apex of the zygomatic arch. While ligaments exist along the upper margin of the zygomatic arch as well, they are not as robust as those below and do not pose significant resistance when pulled upward.^14^ In fact, the threads might catch onto these ligamentous structures and, when pulled upwards, the skin tissues connected here tend to move naturally along with the movement of ligamentous tissues, staying better positioned and not sagging downward compared to looser tissues unconnected to the ligaments.

Following the application of local anesthesia at the entry point, the thread is inserted supra‐superficially into the temporal fascia layer through an 18G needle puncture. Carefully position the thread above the superficial temporal layer, ensuring it is not placed too deep. Following this plane will naturally lead the cannula into the subgaleal plane inside the hairline (Videos S3 and S4).

To achieve optimal effectiveness, the thread should be inserted to some length. The end of the cannula should progress beyond the superior temporal line, a ligamentous structure that forms the upper margin of the temporal fossa, allowing the thread's bulge to adequately pull tissues toward the temple area (Figure 2B,C).

However, relying solely on this lateral cheek lifting (focused on fixed fascia for core‐lifting) has limitations. To address marionette lines and nasolabial folds in older age, additional vectors need to be employed.

**FIGURE 3:**
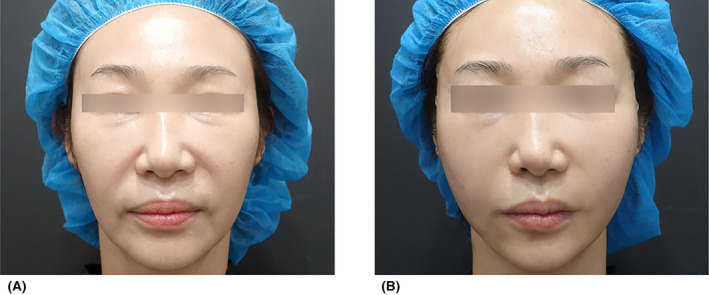
A 43‐year‐old female patient visited with complaints of forearm wrinkles and marionette lines. Bidirectional thread (Epiticon BI, Jetema Inc., Korea) measuring 10 cm in length were used, and a total of eight lines were inserted using the crisscross technique, with four lines inserted on each side. The images depict the patient's appearance just before the procedure (A) and 1 month after the procedure (B).

Case 1 A female patient, aged 43, presented with concerns regarding forearm wrinkles and marionette lines. Multidirectional and bidirectional threads (Epiticon Original and BI, Jetema Inc., Korea) measuring 10 cm in length were utilized. Using the crisscross technique, a total of 8 lines were inserted, with four lines inserted on each side of the face (Figure 3).

## 4 BUCCAL CHEEK & MARIONETTE LINE OBLIQUE LIFTING (PERIPHERAL LIFTING)

**FIGURE 4:**
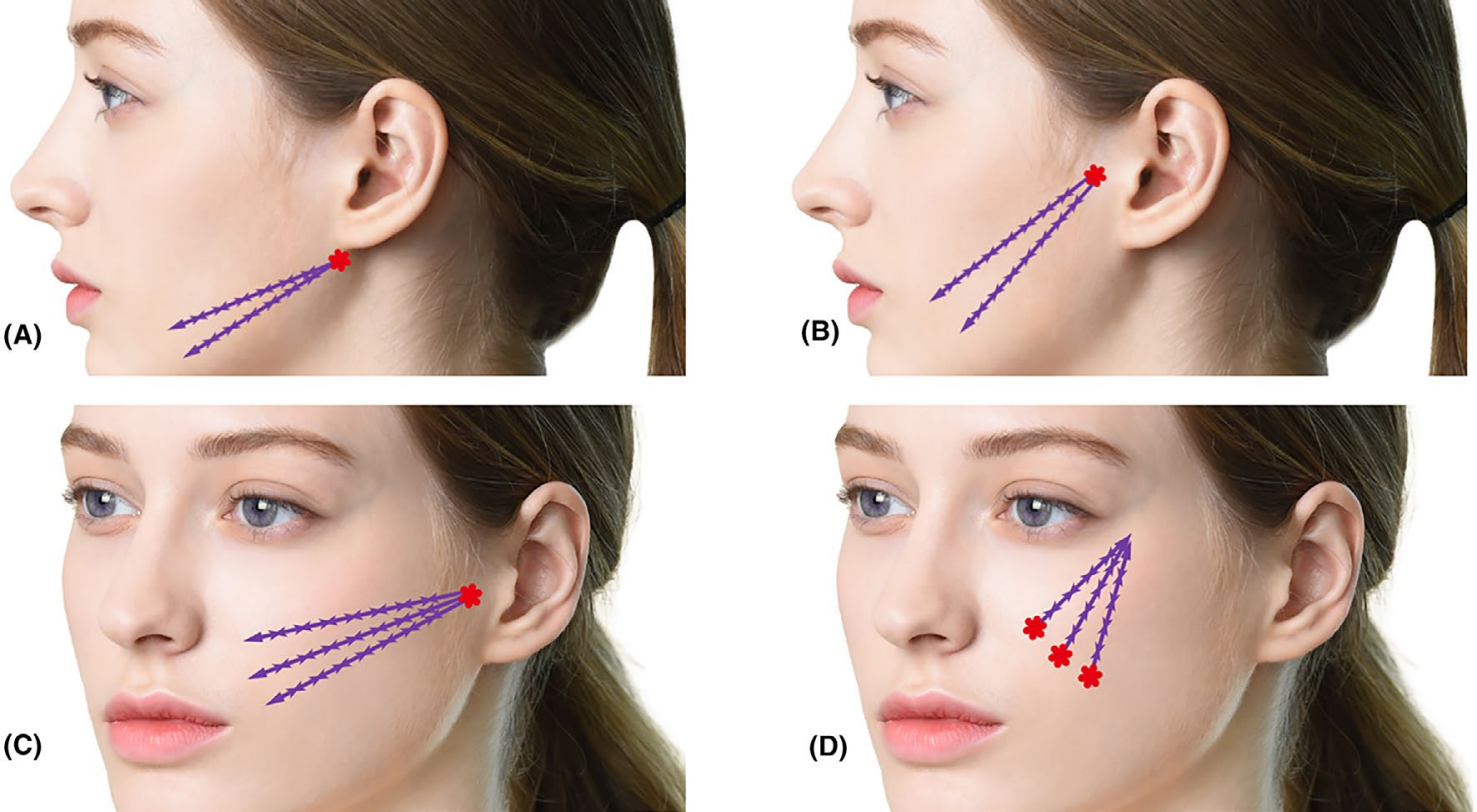
(A) demonstrates the Buccal cheek & marionette line oblique lifting technique, addressing jowling‐induced wrinkles above the mouth corners that tend to follow a vertical direction toward the ears. This method involves effectively pulling and suspending the more robust tissues toward the ear area and requires marking the entry point in front of the tragus. (B) showcases the Mandible border line oblique lifting vector, aiming to refine the mandible border line and provide additional correction for the marionette line. It is crucial during thread insertion to maintain an appropriate layer to prevent skin entrapment or irregularities due to the insertion plane's proximity to the skin. If such issues arise, retracting the cannula by grasping the thread at the rear helps readjust to the suitable layer. The entry point for this technique is located beneath the earlobe. Addressing nasolabial fold treatment becomes difficult when maneuvering the cannula around the malar eminence due to challenges with mouth movement and shallow treatment layers; utilizing Lore's fascia, precise cannula insertion at the tragus‐cheek border for the (C) and targeting the superficial fat layer above the SMAS offer potential solutions. In the (D) in cases where individuals have a band‐like appearance and bulge at the start of their laugh lines near the nose wings, pulling the area adjacent to the nose wings is crucial, though it may challenge the treatment plane, suggesting retrograde insertion from the nasolabial fat to the lateral orbital thickening as a preferred fixation point, using unified PDO threads, and tunneling the cannula beneath pronounced midcheek grooves to facilitate comfortable thread passage.

Jowling, resulting in wrinkles above the corners of the mouth, follows a vertical direction toward the ears. Therefore, effectively addressing this involves pulling and suspending the firmer tissues toward the ear area (Figure 4A,B). The insertion points are located either in front of the tragus or below the earlobe.

As the insertion progresses forward, maintaining an adequate layering is crucial to ensure that the insertion plane of the thread stays at an appropriate distance from the skin. This prevents skin entrapment or irregularities. If such occurrences arise during the insertion process, grasping the thread at the rear allows the entire cannula to be retracted, facilitating the identification of the appropriate layer (Video S4).

## 5 NASOLABIAL FOLD LIFTING (ADDITIONAL CONTOURING)

When addressing prominent nasolabial folds, it is most effective for the vector of the thread to align perpendicular to the fold lines. However, when utilizing the firm tissues near the hairline as an adhesion point, such as in the temporal area, navigating toward the laugh lines becomes challenging. The cannula must bend as it passes the protruding malar eminence and enters towards the front, making it difficult to maneuver realistically (Figure 4C). Additionally, maintaining a consistent insertion plane of the cannula can be problematic around the bent area of the malar eminence, potentially causing a shallow treatment layer.

Moreover, as the thread progresses toward the laugh lines, passing through the origin of the muscles near the malar eminence, it might hinder the movement that raises the corners of the mouth and can accentuate the prominence of the malar eminence, especially in individuals with well‐developed cheekbones.

Therefore, it is advisable to use Lore's fascia, located just in front of the tragus of the ear but slightly lower, as the adhesion point. Careful insertion of the cannula through the entry point made at the border of the tragus and cheek allows for the passage through the SMAS layer without concerns about the superficial temporal artery.^15^ It is essential to avoid entangling the deeper tissues, including the SMAS, extensively with the thread's bulge, as this could interfere with the movement of the combined SMAS and muscles. Therefore, targeting the superficial fat layer above the SMAS is crucial. To achieve this, the cannula is advanced along the SMAS toward the laugh lines. However, at the boundary of the lateral and anterior face, the insertion layer is switched to the deep subcutaneous fat layer directly above the SMAS.^16^

Case 2 A female patient, aged 48, presented with concerns about sagging buccal cheek fat, marionette lines, and nasolabial folds. Insertion points were designated at the tragus, and two lines of multidirectional and bidirectional threads (Epiticon Original and BI), each measuring 8 cm in length, were inserted in the direction of the nasolabial fold and marionette lines. A total of eight lines were employed on both sides of the face.

## 6 REVERSAL TECHNIQUE FOR NASOLABIAL FOLD CORRECTION

In such cases, it is preferable to anchor the lateral orbital thickening of the orbital rim as a fixation point for the procedure. The insertion of the thread should be retrograde (from caudal to cephalic), starting from the position of the nasolabial fat (Figure 4D). A frequently used type is the unified PDO thread with lengths ranging from 6 to 8 cm. If the midcheek groove, which crosses the front of the cheek area diagonally, is pronounced, it is recommended to tunnel the cannula through the fibrous tough band under the groove to create ample space for the thread to pass comfortably before insertion (Video S1).

**FIGURE 5:**
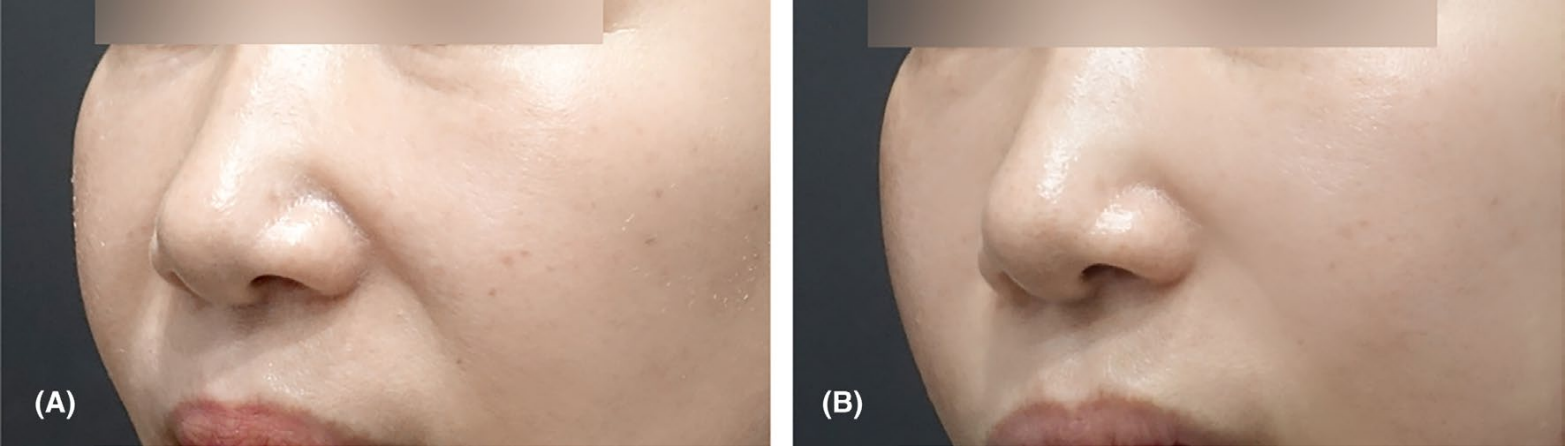
A 53‐year‐old female patient presented with nasolabial fold as her primary complaint. Three entry points were marked above the nasolabial fold, and bidirectional threads (Epiticon BI, Jetema Inc., Korea) measuring 8 cm in length were inserted in the reverse direction for lifting. A total of six threads were used, and the images depict the patient's appearance just before the procedure (A) and 1 month after (B) the procedure.

Case 3 A female patient, aged 53, sought treatment primarily for nasolabial folds. Three entry points were identified above the nasolabial fold, and bidirectional threads (Epiticon BI, Jetema Inc., Korea) measuring 8 cm were inserted in a reverse direction for lifting. A total of six threads were utilized. Please refer to Figure 5 for visual representation.

## 7 DISCUSSION

When selecting absorbable threads for lifting, it is advisable to choose a product that has been properly manufactured, with the shape and design of the thread customized to match the practitioner's specific needs.^17^, ^18^ These threads should have minimal side effects, provide comfort during use, and deliver the desired lifting effects while ensuring an appropriate duration of effectiveness. Moreover, the incorporation of ultrasonography during the procedure aids practitioners in precisely executing thread lifting by providing visual guidance of the anatomical layers during thread insertion.^19^ The retrospective case series conducted by Kim et al.^20^ investigated parotid gland complications resulting from thread lifting procedures. Their study, comprising 703 patients who underwent facial lifting using PDO threads from November 2020 to October 2022, underscored the significance of preoperative ultrasound assessment to locate the parotid gland before thread insertion. The findings revealed that eight patients experienced parotid gland perforation, while one suffered parotid duct injury during this period. Symptoms ranged from tenderness and swelling in six patients to severe swelling and pain in one with duct injury. Nevertheless, conservative treatment led to improvement in all cases, as confirmed by progressive ultrasound findings.^19^ Ultrasound‐guided identification of the parotid gland's position before thread lifting could mitigate the risk of parotid duct injury and enable prompt intervention for delayed symptoms, potentially averting further complications. Complications may arise if the thread is inserted beneath the SMAS layer, potentially resulting in damage to the parotid gland or facial nerves. Additionally, superficial insertion of threads may lead to irregularities or dimpling in the skin.

To address these concerns, fixation threads are employed to maintain the lifted position more effectively and prevent dimpling. It is advantageous to use fixed‐type products such as fixation threads (Epiticon ORIGINAL., Jetema Inc., Korea), which do not require consideration of the patient's facial length. With bi‐directional threads, there are distinct lifting and anchoring regions. This occurs because the direction of the cog is opposite from the midpoint during lifting, causing tissue to gather where the direction of the thread changes. Lower tissues are pulled up while upper tissues descend downwards. However, in the midface area where facial length varies and is relatively short, the cogs serving as anchors may need to be cut. This results in fewer cogs and potentially reduces the duration of effectiveness due to patient discomfort or stress induced by gravity on specific areas.

Fixation threads, unlike conventional uni‐directional or bi‐directional threads, allow both cogs to simultaneously anchor and lift, enabling contouring and fixation. Surgical procedures utilizing fixation thread structures are versatile, particularly when enhancing tissues by gathering during facial lifting procedures. After tissue repositioning, maximizing stabilization in the new position can be achieved with fixation threads.

Beyond mere tensile forces pulling and repositioning the tissue initially, the holding strength of the threads becomes vital.^3^ This strength helps maintain the tissue in the relocated area, withstand loads exerted in the opposite direction of gravity, and prevent the tissues from returning to their original position. Customized planning based on the patient's facial structure and needs, along with the judicious use of appropriate threads, can lead to favorable outcomes.^21^, ^22^, ^23^

In addition to the risks of parotid gland and duct injury and motor nerve paralysis associated with thread lifting, several other complications may arise, necessitating surgical intervention such as the removal of threads and debridement.^24^, ^25^ These complications include thread protrusion, subcutaneous nodules, and infection. Among the most frequently noted complications are persistent pain, thread protrusion, dimpling, sensory abnormalities, and foreign body reactions.^24^, ^25^, ^26^, ^27^, ^28^, ^29^, ^30^ According to a study conducted by Rachel et al.,^28^ there appears to be an increased risk of complications when a greater number of threads are employed in thread lifting procedures.^17^ Although occurrences of chronic inflammatory reactions are infrequent, research underscores the possibility of chronic inflammation being induced in facial soft tissues due to repetitive trauma and micro‐movements between the sutures' barbs and the surrounding tissue.^31^

Even rarer complications, such as iatrogenic superficial temporal artery pseudoaneurysm, have been documented. For instance, Nimii et al.^32^ reported a case where a 27‐year‐old man developed this complication after a midface thread‐lift procedure, presenting with a painless, pulsating soft mass in the pre‐auricular region. Surgical intervention was required, entailing the resection of the pseudoaneurysm and microsurgical reconstruction of the superficial temporal artery. While occurrences are deemed infrequent based on current literature, conducting comprehensive institutional studies with larger case samples is crucial to understand complication rates and preventive measures better. These complications can prolong patient recovery and impact both aesthetic and functional outcomes. Hence, it is vital for practitioners and patients to be informed about these potential complications and carefully weigh the risks and benefits before proceeding with thread lifting procedures.^26^, ^33^

Hong et al.^24^ explored the factors contributing to the longevity of thread lifting. These factors encompassed the materials used for the threads, thread thickness and tensile strength, the configuration and number of cogs for anchoring and holding strength, the orientation of cogs, the depth of thread insertion based on tissue resilience, the selection of suitable lifting vectors and anchoring points according to facial anatomy, the distinctions between flap and string methods, and the expiration date of absorbable threads. Considering all these factors is crucial for practitioners when performing thread lifting procedures to ensure optimal outcomes and patient satisfaction.

This study is subject to certain limitations that warrant consideration. Firstly, the absence of objective assessments to evaluate the efficacy of the thread lifting procedures is noteworthy. While the study demonstrated significant improvements in the correction of the nasolabial fold and marionette line, the lack of objective measures, such as standardized rating scales or quantitative measurements, makes it challenging to precisely determine the extent of improvement or compare the outcomes with other treatment modalities.

In conclusion, it is anticipated that tailored treatment plans and the appropriate selection of threads will enhance the attainment of optimal results for the patient.

## AUTHOR CONTRIBUTIONS

All authors have reviewed and approved the article for submission. **Kyu‐Ho Yi, Soo Yeon Park**: conceptualization. **Soo Yeon Park, Jovian Wan**: writing—original draft Preparation. **Soo Yeon Park, Jovian Wan, Soo‐Bin Kim**: writing—review and editing. **Soo Yeon Park, Kar Wai Alvin Lee, Lisa Kwin Wah Chan, Atchima Suwanchinda, Kashif Ali Samin**: visualization. **Kyu‐Ho Yi, Soo‐Bin Kim**: supervision.

## FUNDING INFORMATION

There is no financial disclosure to report.

## CONFLICT OF INTEREST STATEMENT

I acknowledge that I have considered the conflict of interest statement included in the “Author Guidelines.” I hereby certify that, to the best of my knowledge, that no aspect of my current personal or professional situation might reasonably be expected to significantly affect my views on the subject I am presenting.

## ETHICS STATEMENT

This study was conducted in compliance with the principles set forth inthe Declaration of Helsinki.

## Supporting information


Video S1.

Video S2.

Video S3.

Video S4.

## Data Availability

The data that support the findings of this study are available from the corresponding author upon reasonable request.
